# Folate/homocysteine metabolism and lung cancer risk among smokers

**DOI:** 10.1371/journal.pone.0214462

**Published:** 2019-04-02

**Authors:** Anna Stanisławska-Sachadyn, Joanna Borzyszkowska, Michał Krzemiński, Alicja Janowicz, Rafał Dziadziuszko, Jacek Jassem, Witold Rzyman, Janusz Limon

**Affiliations:** 1 Department of Biology and Genetics, Medical University of Gdańsk, Gdańsk, Poland; 2 Department of Molecular Biotechnology and Microbiology, Gdańsk University of Technology, Gdańsk, Poland; 3 Department of Probability and Biomathematics, Gdańsk University of Technology, Gdańsk, Poland; 4 Department of Thoracic Surgery, Medical University of Gdańsk, Gdańsk, Poland; 5 Department of Oncology and Radiotherapy, Medical University of Gdańsk, Gdańsk, Poland; 6 Gdańsk Branch of the Polish Academy of Sciences, Gdańsk, Poland; Sapporo Ika Daigaku, JAPAN

## Abstract

**Background:**

Folate and homocysteine are involved in DNA synthesis and methylation processes, which are deregulated during carcinogenesis.

**Objectives:**

The aim of this study was to assess the relationship between folate/homocysteine concentrations, the functional polymorphisms of folate/homocysteine genes and lung cancer risk among cigarette smokers.

**Study design:**

The study included 132 lung cancer patients and 396 controls from northern Poland, matched by sex, age and smoking status. The median cigarette pack-years of smoking among both cases and controls was 30.0. Serum, red blood cell (RBC) folates and serum homocysteine concentrations were measured. The genotypes in selected polymorphic sites of the *MTHFR*, *CBS*, *SHMT1*, *MTHFD1*, *MTRR*, *MTR*, *TYMS DHFR*, *TCN2*, and *SLC19A1* genes were determined. All study participants underwent scanning with low-dose computed tomography.

**Results:**

Serum folate concentrations above the median (> 17.5 nmol/l among the healthy controls) were associated with an increased lung cancer risk (odds ratio [OR], 1.54, 95% confidence intervals [CI], 1.04–2.29, *P* = 0.031). An analogous trend was observed when the population was analysed after subdivision according to RBC folate concentrations, that is, above a value of 506.5 nmol/l (OR, 1.53; 95% CI, 0.95–2.47; *P* = 0.084). Additionally, in a subset of women, an increased risk of lung cancer development was associated with the *SLC19A1* c.80AA genotype (c.80AA versus GG OR, 3.14; 95% CI, 1.32–7.46; *P* = *P* = 0.010).

**Conclusion:**

These results suggest that, in the population consisting of heavy smokers, high folate levels add to the cancerogenic effect of smoking.

## Introduction

Lung cancer accounts for the highest number of cancer deaths globally [1], with cigarette smoking remaining the most prominent risk factor [2].

**Fig 1:**
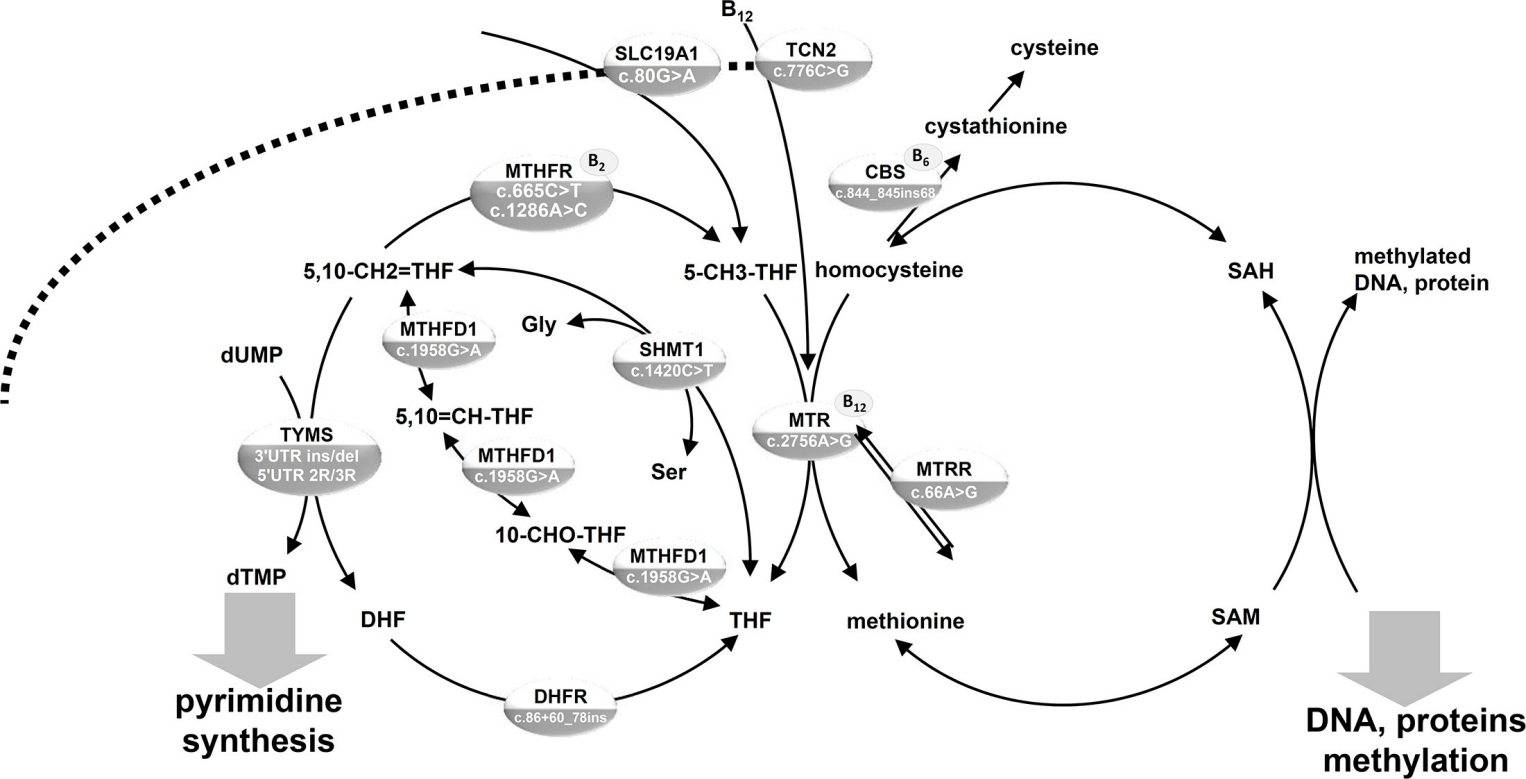
Schematic representation of C1 metabolism. DNA polymorphisms determined in lung cancer case-control study population from northern Poland. C1, methyl group; CBS, cystathionine-beta-synthase; DHFR, dihydrofolate reductase; HCOOH, formate; MTHFD1, methylenetetrahydrofolate dehydrogenase-methenyltetrahydrofolate cyclohydrolase-formyltetrahydrofolate synthetase (soluble); MTHFR, 5,10-methylenetetrahydrofolate reductase; MTR, methionine synthase; MTRR, methionine synthase reductase; SAM, S-adenosylmethionine; SAH, S-adenosylhomocysteine; SLC19A1, solute carrier family 19 (reduced folate transporter), member 1; SHMT1, serine hydroxymethyltransferase 1 (soluble); TCN2, transcobalamin; THF, tetrahydrofolate; TYMS, thymidylate synthase; 5-CH3-THF, 5-methyltetrahydrofolate; 5-CHO-THF, 5-formyltetrahydrofolate; 5,10-CH2 = THF, 5,10- methylenetetrahydrofolate; 5,10 = CH-THF, 5,10- methenyltetrahydrofolate; 10-CHO-THF, 10-formyltetrahydrofolate.

Folate (vitamin B_9_) plays a pivotal role in cell metabolism by providing metabolites necessary for DNA synthesis and repair, as well as for methylation of DNA, proteins and lipids. While reduced folates are crucial C1 (one-carbon, methyl group) cycle intermediates, several enzymes involved in their metabolism require other cofactors from the B-vitamin group, such as B_12_ (Fig 1). Moreover, 5-methyl-tetrahydrofolate (5-methyl-THF) acts as a methyl group donor to remethylate homocysteine to methionine, representing an intermediate necessary for methylation processes.

Folate deficiency is associated with a higher incidence of neural tube defects (NTDs) and it has been proved that periconceptional folic acid supplementation decreases the NTD rate by 70% [3]. This beneficial effect of folic acid supplementation has led to the introduction of mandatory folic acid food fortification in the United States, Canada, Chile, Costa Rica and South Africa [4]. In addition, high homocysteine concentration is a marker of increased stroke risk, and this has been suggested to result from long-term inadequate B-vitamins levels and endothelial dysfunction. There is accumulating evidence that folic acid interventions aimed at lowering homocysteine levels decrease the incidence of stroke [5].

Despite beneficial effects of folic acid supplementation in some malignancies, there are some concerns related to the potential impact of these interventions, or even folate status, on cancer progression. High folate levels may enable the progression of neoplastic cells to cancer via the stimulation of DNA synthesis and cell proliferation [6]. In contrast, inadequate folate levels in neoplastic cells may be associated with the inhibition of DNA synthesis, and thus may lead to the cell growth arrest [6]. However, suboptimal folate levels are also associated with a higher rate of DNA breaks, mutations and alterations in DNA methylation patterns; thus potentially triggering transformation of normal to neoplastic cells.

The combined results of two randomised, double-blind, placebo-controlled clinical trials—the Norwegian Vitamin Trial and the Western Norway B Vitamin Intervention Trial [7]—indicated that folic acid and vitamin B_12_ supplementation was associated with increased lung cancer incidence. However, in another study from Finland, higher serum B_6_ levels in men were associated with decreased risk of lung cancer, while serum folate levels were not associated to altered lung cancer risk [8]. Interestingly, a large European study comprising 899 lung cancer cases and 1770 controls showed that high levels of both vitamin B_6_ and methionine were negatively correlated with lung cancer risk, whereas among ex- and current smokers higher folate levels were associated with a lower lung cancer risk [9]. Further, the mean serum folate level was found to be lower in lung cancer cases as compared to controls [10].

Since several genetic polymorphisms are known to be modulators of folate/homocysteine status, establishing their association with lung cancer risk may elucidate the role of C1 metabolism in lung carcinogenesis. The associations between both well-known, functional polymorphisms: the *MTHFR* c.665C>T [11–23], *MTHFR* c.1286A>C [11, 14–20, 22, 23], *MTR* c.2756A>G (14–18), *MTRR* c.66A [14, 15, 17, 19], *SHMT1* c.1420C>T [14, 24], *TYMS* 6bp ins/del polymorphism within the 3’UTR (untranslated region) [25], *TYMS* 5’UTR 2R/3R [15, 25] and other variants within the *MTHFR* [18, 26], *TYMS* [18], *MTHFD1* [18], *CBS* [19], *SLC19A1* [19], *SHMT1* [24] and *MTRR* gene [26] and lung cancer risk have been evaluated.

The aim of this study was to investigate whether folate metabolism may add to the lung cancer risk in a large, homogenous population of long-term smokers from northern Poland. Specifically, we determined an association between the risk, serum folate, red blood cell (RBC) folate and homocysteine levels or several known functional polymorphisms in the C1 metabolism genes.

## Materials and methods

### Study design

A total of 1080 blood samples were collected between October 2009 and March 2011 during the Pomeranian Lung Cancer Screening Program performed at the Medical University of Gdańsk, which offered low-dose computed tomography (LDCT) examination for more than 8600 current or former smokers ranging from 50 to 75 years of age. Among the participants of the screening program 16 lung cancer cases were diagnosed. Therefore, between April 2010 and November 2011, blood samples from 116 incidentally detected lung cancer patients before any therapeutic intervention, who did not participate in the screening program, were added to the study, on the condition each case could have been randomly matched to three controls for age, sex and pack-years of smoking. All controls were selected from the participants of the Pomeranian Lung Cancer Screening Program.

The criteria for matching cases and controls were as follows: sex; pack-years with an accuracy of 10 pack-years, and if this criterion could not be fulfilled, an accuracy of 30 pack-years; an age matching with an accuracy of 1 year, and if this criterion could not be fulfilled, an accuracy of 2 years. The matching criteria were fulfilled by 132 cases and 396 controls.

The study was approved by the Ethical Committee of Medical University of Gdańsk, Poland (consent number: NKEBN/42/2009). All procedures performed were in accordance with the 1964 Helsinki declaration and its later amendments. Informed consent was obtained from all individual participants included in the study.

### Biochemical analyses

Serum and RBC folate concentrations were determined using chemiluminescent microparticle folate binding protein assay (Abbott, Diagnostics Division, Ireland), while serum homocysteine concentrations were tested by immunonephelometric assay (Siemens, Germany). RBC folate concentrations were determined from the whole blood and were corrected for the hematocrit values. RBC folate concentrations corrected for serum folate concentrations were calculated according to Abbott’s protocol. For 36 cases and 3 controls the measurements of serum folate and serum homocysteine were determined from samples frozen in -80°C prior to analyses. For 33 cases the measurements of RBC folates were determined from whole blood diluted in 1% ascorbic acid at a proportion of 1:10, and frozen in -80°C prior to analyses, using previously validated modified chemiluminescent microparticle folate binding protein assay (Abbott), the mean value from two measurements was taken as the final result. Briefly, 100 μl of Folate RBC Lysis Diluent L2 (Abbott) and 100 μl of whole blood sample diluted 1:10 in 1% ascorbic acid were mixed, next Abbott procedure was followed, samples were read using Folate assay. In order to assess the potential impact of sample pre-treatment, we performed the measurements of metabolites from differently processed aliquots of randomly selected samples and we analysed the correlations between the results (S1 Fig Supplementary Information). These analyses demonstrated that the different pre-treatment conditions were unlikely to have impacted the measurements of metabolites. All the other samples were transferred to the laboratory immediately after collection. The analyses were performed by the Central Laboratory of Clinical Centre of Medical University of Gdańsk, Poland.

### Genotyping

DNA for genetic analyses was isolated from buffy coats using QIAamp DNA Blood Midi Kit (Qiagen). The population was genotyped in the *MTHFR* c.665C>T (rs1801133), *MTHFR* c.1286A>C (rs1801131), *SHMT1* c.1420C>T (rs1979277), *MTHFD1* c.1958G>A (rs2236225), *MTRR* c.66A>G (rs1801394), *MTR* c.2756A>G (rs1805087), *TYMS* 3'UTR ins/del (rs202243265, rs11280056, rs16430), *TCN2* c.776C>G (rs1801198) and *SLC19A1* c.80G>A (rs61510559) polymorphisms using 5’ Nuclease Real-Time PCR assay on a Light Cycler 480 system (Roche). Individual PCR amplification reactions (20 μl) were composed of 2 μl sample DNA, 1xTaqMan Universal PCR Master Mix, No AmpEraseR UNG (Roche), forward and reverse primers and two allele-specific probes. The probes were synthesised by Applied Biosystems. Each plate was set up with a no template control and positive controls representing all genotype classes. Some primers/probes sequences were obtained from published assays [27–33] and the SNP500 Cancer website (http://snp500cancer.nci.nih.gov/cgfseq/pages/snp500.do).

Population was genotyped in the *CBS* c.844_845ins68, *TYMS* 5'UTR 2R/3R, *DHFR* c.86+60_78ins22 polymorphisms using PCR, followed by agarose gel electrophoresis.

Primers, probes sequences and concentrations and genotyping conditions are listed in S1 Table.

### Statistical analyses

Descriptive analyses of the study variables included medians and percentiles for continuous variables and proportions for categorical variables. Deviations from the Hardy-Weinberg equilibrium for the genotypes were assessed by χ2 analysis. Correlations between the metabolite values were calculated using Pearson statistics.

To calculate odds ratios (ORs) with 95% confidence intervals (CI) of lung cancer conditional logistic regression (proc logistic in SAS), conditioning on individual case sets, were used. The population was subdivided into groups defined by the upper and the lower half of distribution of biochemical variables. Medians were selected based on the distributions among the healthy controls. Subjects in the lower half of distribution were selected as a reference group. All available biochemical variables were transformed to categorical variables. In analyses involving gene polymorphisms, homozygous carriers of the major allele were selected as a reference group. This was followed by calculations of *p*-values for the trends by applying score defined by a following natural numbers for each genotype (0- homozygous carriers of the major allele, 1-heterozygotes, 2- homozygous carriers of the minor allele). Next, the OR of lung cancer for each genotype versus the other allele carriers were evaluated. As four test were calculated to evaluate each SNP, the Bonferroni method was used to correct the results for multiple comparisons.

The OR of lung cancer associated with the *SLC19A1* c.80G>A genotypes were calculated using unconditional logistic regression controlling for the matching factors, separately for men and women and in either lower or upper serum folate and homocysteine groups (groups were defined as described above).

Differences in distributions of serum folate, RBC folate, RBC folate corrected and homocysteine concentrations between the genotypes in groups defined by disease status and sex were tested using the Kruskal–Wallis test or the Wilcoxon rank-sum tests, when appropriate. *P*-values ≤0.05 were considered statistically significant. The extreme values: serum folate of 800.5 and 565.4 nmol/l were excluded from those analyses, as indicated in table footnotes when necessary.

Statistical analyses were calculated using SAS 9.3 (NC, USA).

## Results

The characteristics of the study group are presented in Table 1. The study included 132 lung cancer patients and 396 controls from northern Poland. Women accounted for 46% of participants. Among both the cases and controls the median cigarette pack-years of smoking was 30.0. The median age of both cases and controls was 62.9. The majority of lung cancer cases were diagnosed with adenocarcinoma (51%) and with squamous cell cancer (36%). The majority of subjects were not supplemented with B-vitamins. Serum folates and homocysteine as well as RBC folate and homocysteine were negatively correlated (r = -0.19; N = 522; *P* <.0001 and r = -0.19; N = 368; *P* = 0.0002, respectively) while serum folate and RBC folate levels were positively correlated (r = 0.56, N = 371, *P* <.0001).

**Table 1:** Characteristics of participants in lung cancer case-control study population from northern Poland.

Variable		Lung cancer cases (N = 132)	Controls (N = 396)
Sex	Women	61 (46%)	183 (46%)
	Men	71 (54%)	213 (54%)
Age	Mean, SD	62.8 (6.2)	62.7 (6,1)
	Median (Min-Max)	62.9 (48.6–77.1)	62.9 (49.2–75.9)
Pack-years of smoking^a^	Mean, SD	32.2 (15.0)	32.3 (11.1)
	Median (Min-Max)	30.0 (1–75)	30.0 (7.5–80)
Source of cases	Screening	16 (12%)	
	Incidental	116 (88%)	
Cancer subtype	Adenocarcinoma	67 (51%)	
	Squamous Cell Cancer	47 (36%)	
	Small Cell Lung Cancer	2 (2%)	
	Large Cell Carcinoma	8 (6%)	
	Other	8 (6%)	
Cancer clinical stage	In situ	1 (1%)	
	1a	40 (30%)	
	1b	26 (20%)	
	2a	30 (23%)	
	2b	13 (10%)	
	3a	21 (16%)	
	4	1 (1%)	
Serum folates nmol/l^b^	Median (25^th^-75^th^ percentile), N	19.3 (14.3–24.3), 132	17.5 (12.9–23.1), 394
RBC folates nmol/l	Median (25^th^-75^th^ percentile), N	559.7 (401.5–721.9), 93	506.5 (383.3–677.4), 279
RBC folates corrected nmol/l	Median (25^th^-75^th^ percentile), N	524.7 (378.4–684.4), 93	475.9 (367.0–642.5), 278
Homocysteine μmol/l	Median (25^th^-75^th^ percentile), N	15.7 (11.4–18.6), 129	14.7 (12.0–18.6), 395
Supplementation	No	87 (66%)	268 (68%)
	Yes	12 (9%)	123 (31%)
	No data	33 (25%)	5 (1%)
Supplementation with B vitamins	No	97 (73%)	362 (91%)
	Yes	2 (2%)	28 (7%)
	No data	33 (25%)	6 (2%)

^a^a pack-year is defined as one pack of cigarettes smoked per day for one year.

^b^Two outlying extreme values were excluded from the analyses: serum folate = 800.5; 565.4 nmol/l.

**Fig 2:**
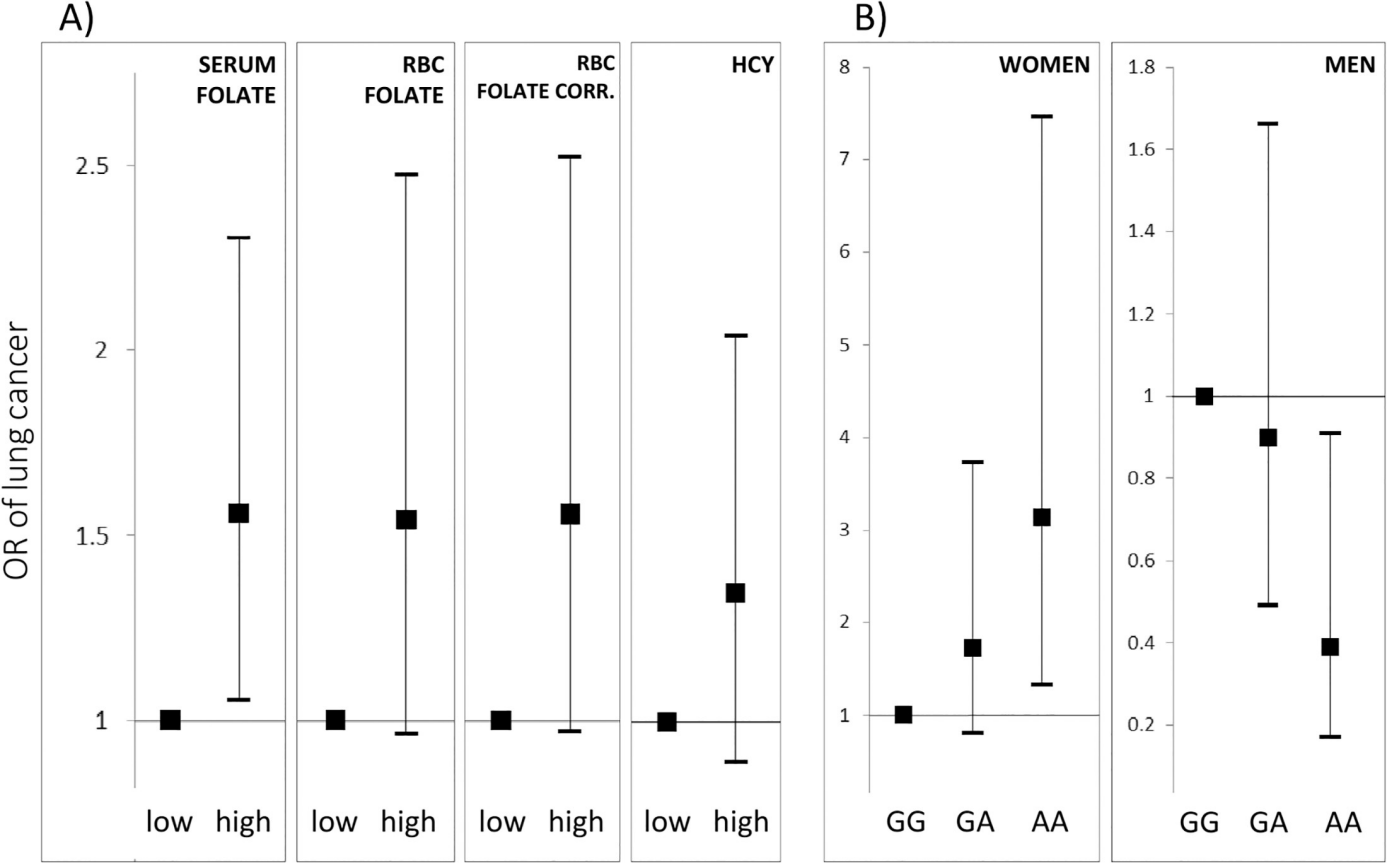
Odds ratios of lung cancer occurrence in relation to A) serum folate, RBC folate, RBC folate corrected and homocysteine levels B) the *SLC19A1* c.80A>G genotypes among women and men, in case-control study population from northern Poland.

No significant differences between serum folate, RBC folate or homocysteine between the cases and controls (Table 1) were found. Subsequently, the population was subdivided by median values (among healthy subjects) of serum folate, RBC folate, RBC folate corrected, and homocysteine measures (Table 2, Fig 2). The risk of lung cancer incidence correlated with high levels of serum folate (OR = 1.54, 95% CI: 1.04–2.29, *P* = 0.031). An analogous trend was observed after subdivision according to median RBC folate concentrations (OR = 1.53, 95% CI: 0.95–2.47, *P* = 0.084) and RBC folate concentrations corrected for serum folates (OR = 1.51, 95% CI: 0.93–2.43, *P* = 0.093). No differences in lung cancer risk were found when subdivision was applied with regard to homocysteine levels (Table 2).

**Table 2:** Odds ratios of lung cancer occurrence in relation to serum folate, RBC folate and homocysteine levels, in study population from northern Poland, calculated by conditional logistic regression with adjustment for sex, age and pack-years of smoking.

Variable		Lung cancer cases (%)	Controls (%)	OR (95% CI), *P*
Serum folates nmol/l	≤17.5	52 (39.4)	200 (50.5)	1 (Ref)
	>17.5	80 (60.6)	196 (49.5)	1.54 (1.04–2.29), P = 0.031
RBC folates nmol/l	≤506.5	37 (39.8)	140 (50.2)	1 (Ref)
	>506.5	56 (60.2)	139 (49.8)	1.53 (0.95–2.47), P = 0.084
RBC folates corrected nmol/l	≤474.8	37 (39.8)	139 (50.0)	1 (Ref)
	>474.8	56 (60.2)	139 (50.0)	1.51 (0.93–2.43), P = 0.0934
Homocysteine μmol/l	≤ 14.7	56 (43.4)	203 (51.4)	1 (Ref)
	>14.7	73 (56.6)	192 (48.6)	1.41 (0.93–2.14), P = 0.1021*

*homocysteine measures for 3 cases were not available, thus 9 matched subjects from the control group were excluded from analyses as uninformative data

The association between the genotypes in the C1 metabolism-involved genes and the lung cancer risk was assessed for the population as a whole and in the subgroups defined by sex. The more frequent homozygous genotype was selected as the reference for the step-effect of less common allele increase. All analysed genotypes were in accordance with the Hardy-Weinberg equilibrium. The distributions of biochemical variables defined by genotypes under the study are presented in S2 and S3 Tables.

No significant association between the *MTHFR* c.665C>T polymorphism and lung cancer risk was found, although a trend towards lower risk of lung cancer among T allele carriers as compared to the CC genotype was observed (OR = 0.68, 95% CI: 0.45–1.01, *P* = 0.057, Table 3). The *TYMS* 3’UTR del/del genotype was associated with a lower lung cancer risk as compared to the ins/ins genotype (OR = 0.33, 95% CI: 0.11–0.96, *P* = 0.041, Table 3). This association was not significant, though, after correction for multiple comparisons.

**Table 3:** Odds ratios of lung cancer occurrence in relation to genotypes in the C1 metabolism genes, in study population from northern Poland.

Polymorphism	Genotype	Lung cancer cases (% of genotype carriers)	Controls (% of genotype carriers)	OR (95% CI), *P*
rs1801133 *MTHFR* c.665C>T	CC	65 (49)	158 (40)	1 (Ref)
	CT	55 (42)	199 (50)	0.66 (0.43–1.01), *P* = 0.056
	TT	12 (9)	39 (10)	0.75 (0.37–1.51), *P* = 0.423
				*P* for trend = 0.120
	TvsCC(Ref)			0.68 (0.45–1.01), *P* = 0.057
	TTvsC(Ref)			0.92 (0.47–1.80), *P* = 0.801
rs1801131 *MTHFR* c.1286A>C	AA	69 (52)	193 (49)	1 (Ref)
	AC	49 (37)	169 (43)	0.80 (0.53–1.23), *P* = 0.314
	CC	14 (11)	34 (9)	1.16 (0.59–2.29), *P* = 0.665
				*P* for trend = 0.818
	CvsAA(Ref)			0.87 (0.59–1.29), *P* = 0.482
	CCvsA(Ref)			1.27 (0.65–2.45), *P* = 0.484
*CBS* c.844_845ins68	DD	110 (83)	343 (87)	1 (Ref)
	I allele	22 (17)	53 (13)	1.32 (0.75–2.31), *P* = 0.338
rs1979277 *SHMT1* c.1420C>T	CC	53 (40)	164 (41)	1 (Ref)
	CT	61 (46)	186 (47)	1.02 (0.66–1.57), *P* = 0.935
	TT	18 (14)	46 (12)	1.22 (0.64–2.31), *P* = 0.546
				*P* for trend = 0.618
	TvsCC(Ref)			1.06 (0.70–1.59), *P* = 0.794
	TTvsC(Ref)			1.21 (0.67–2.18), *P* = 0.535
rs2236225 *MTHFD1* c.1958G>A	GG	39 (30)	131 (33)	1 (Ref)
	GA	63 (48)	182 (46)	1.15 (0.74–1.80), *P* = 0.530
	AA	30 (23)	83 (21)	1.21 (0.70–2.09), *P* = 0.496
				*P* for trend = 0.470
	AvsGG(Ref)			1.17 (0.77–1.78), *P* = 0.463
	AAvsG(Ref)			1.11 (0.69–1.78), *P* = 0.669
rs1801394 *MTRR* c.66A>G	GG	50 (38)	119 (30)	1 (Ref)
	GA	58 (44)	190 (48)	1.09 (0.63–1.87), *P* = 0.768
	AA	24 (18)	87 (22)	1.50 (0.87–2.60), *P* = 0.148
				*P* for trend = 0.115
	AvsGG(Ref)			0.70 (0.47–1.06), *P* = 0.095
	AAvsG(Ref)			0.79 (0.48–1.30), *P* = 0.359
rs1805087 *MTR* c.2756A>G	AA	91 (69)	245 (62)	1 (Ref)
	AG	36 (27)	133 (34)	0.73 (0.47–1.13), *P* = 0.162
	GG	5 (4)	18 (5)	0.75 (0.27–2.10), *P* = 0.587
				*P* for trend = 0.178
	GvsAA(Ref)			0.73 (0.48–1.12), *P* = 0.148
	GGvsA(Ref)			0.83 (0.30–2.28), *P* = 0.710
rs202243265 rs11280056 rs16430 *TYMS* 3'UTR ins/del	II	67 (51)	179 (45)	1 (Ref)
	ID	61 (46)	184 (46)	0.88 (0.59–1.33), *P* = 0.547
	DD	4 (3)	33 (8)	0.33 (0.11–0.96), *P* = 0.041
				*P* for trend = 0.079
	3*DvsII(Ref)			0.80 (0.53–1.19), *P* = 0.261
	3*DDvsI(Ref)			0.35 (0.12–1.00), *P* = 0.050
*TYMS* 5'UTR 2R/3R	2R/2R	32 (24)	72 (18)	1 (Ref)
	2R/3R	66 (50)	205 (52)	0.716 (0.43–1.18), *P* = 0.189
	3R/3R	34 (26)	119 (30)	0.62 (0.34–1.12), P = 0.112
				*P* for trend = 0.120
	5' 3Rvs2R2R(Ref)			0.69 (0.42–1.11), *P* = 0.125
	5' 3R3R vs2R(Ref)			0.80 (0.51–1.26), *P* = 0.333
*DHFR* c.86+60_78ins22	II	36 (27)	119 (30)	1 (Ref)
	ID	72 (55)	198 (50)	1.22 (0.76–1.95), *P* = 0.414
	DD	24 (18)	79 (20)	1.01 (0.56–1.80), *P* = 0.985
				*P* for trend = 0.886
	DvsII(Ref)			1.15 (0.74–1.79), *P* = 0.541
	DDvsI(Ref)			0.89 (0.54–1.48), *P* = 0.659
rs1801198 *TCN2* c.776C>G	CC	39 (30)	125 (32)	1 (Ref)
	CG	64 (48)	183 (46)	1.12 (0.71–1.75), *P* = 0.634
	GG	29 (22)	88 (22)	1.05 (0.61–1.82), *P* = 0.850
				*P* for trend = 0.810
	GvsCC(Ref)			1.10 (0.72–1.67), *P* = 0.670
	GGvsC(Ref)			0.99 (0.62–1.58), *P* = 0.952
rs61510559 *SLC19A1* c.80G>A	GG	35 (27)	115 (29)	1 (Ref)
	GA	68 (52)	191 (48)	1.17 (0.73–1.87), *P* = 0.514
	AA	29 (22)	90 (23)	1.06 (0.60–1.88), *P* = 0.834
				*P* for trend = 0.803
	AvsGG(Ref)			1.14 (0.72–1.78), *P* = 0.574
	AAvsG(Ref)			0.96 (0.60–1.54), *P* = 0.857
rs61510559 *SLC19A1* c.80G>A Women	GG	11 (18)	57 (31)	1 (Ref)
	GA	31 (51)	95 (52)	1.72 (0.80–3.73), *P* = 0.168
	AA	19 (31)	31 (17)	3.14 (1.32–7.46), *P* = 0.010
				*P* for trend = 0.009
	AvsGG (Ref)			2.08 (1.00–4.31), *P* = 0.050
	AAvsG (Ref)			2.16 (1.12–4.17), *P* = 0.022
rs61510559 *SLC19A1* c.80G>A Men	GG	24 (34)	58 (27)	1 (Ref)
	GA	37 (52)	96 (45)	0.90 (0.49–1.66), *P* = 0.740
	AA	10 (14)	59 (28)	0.39 (0.17–0.91), *P* = 0.030
				*P* for trend = 0.040
	AvsGG (Ref)			0.73 (0.40–1.31), *P* = 0.284
	AAvsG (Ref)			0.42 (0.20–0.88), *P* = 0.022

Sex-stratified analyses revealed that the *SLC19A1* c.80G>A polymorphism had different associations with lung cancer risk among men and women (Table 3, Fig 2). In women, the *SLC19A1* c.80AA genotype was associated with a higher risk of lung cancer as compared to the GG genotype (OR = 3.14, 95% CI: 1.32–7.46, *P* = 0.01). In men, inversely, the *SLC19A1* c.80AA genotype was associated with a protection against lung cancer as compared to the GG genotype (OR = 0.39, 95% CI: 0.17–0.91, *P* = 0.03), but this association was no longer significant after correction for multiple comparisons. In women, the risk of lung cancer positively correlated with increasing number of A allele (*P* for trend = 0.009), while in men, it correlated negatively with increasing number of A allele (*P* for trend = 0.040).

In order to determine whether the above association might be influenced by serum folate or homocysteine levels, the study population was further divided into groups defined by sex and by either low/high halves of folate or homocysteine distributions, the association between the *SLC19A1* c.80G>A genotypes and the lung cancer risk was assessed within each group. The *SLC19A1* c.80AA genotype presented higher risk in women and lower risk in men, as compared to the GG genotype, regardless of the metabolite halves within which analyses were conducted (data not presented). The prevailing number of P-values did not reach significance, which may be due to low numbers of subjects within subgroups.

The conditional logistic regression analyses to calculate odds of lung cancer development in relation to the *SLC19A1* c.80G>A genotypes with correction for serum folate levels, in subgroups defined by sex, were performed. Crude and adjusted ORs for the association between lung cancer occurrence and the *SLC19A1* c.80G>A genotypes were not substantially different. The significance of results did not change after adjustment for folate levels.

## Discussion

Our data suggest that serum folate status influence the lung cancer risk among smokers in the northern Polish population. Specifically, more lung cancer cases were found in subjects with serum folate levels above a value of 17.5 nmol/l. This observation corresponds to the report from the Norwegian Vitamin Trial and Western Norway B Vitamin Intervention Trial [7], which found a higher hazard ratio of lung cancer incidence in subjects who were administered folic acid as compared to those who were not. On the contrary, however, Johansson *et al*. [9] found that higher folate levels were associated with lower lung cancer risk, while Hartman *et al*. [8] reported no association. This discrepancy may be due to differences in the structures of populations, folate levels distributions in each population, ethnicity or tobacco exposure. Interestingly, heavy smokers were predominant in our study while the association reported by Johansson *et al*. [9] included the significant fraction of former smokers. Our and Hartman *et al*. [8] cohorts markedly differ in average folate levels. Specifically, subjects with serum folate levels below 6 ng/ml constituted 90% of the Finnish population but only 26% of that we report herein. Consequently, the majority of the Finnish cohort had folate levels lower than those we associated with increased lung cancer risk. Next, the contribution of smokers in two intervention trials: WAFACS [34] and HOPE-2 [35], which reported no effect of supplementation with folic acid, vitamin B_12_ and B_6_ on lung cancer incidence, was low (11.9%; 11.5%, respectively) and subjects mainly came from the countries with mandatory folate fortification.

Folates have been postulated to have a potentially complex impact on carcinogenesis. Folate prevents mutagenesis in normal cells, but after initial DNA lesions occur, increased folate levels may shift the balance towards progression of neoplastic cells to cancer due to an increased synthesis of nucleic acids [6]. Moreover, C1 metabolism, via its impact on SAM levels (Fig 1), has been postulated to effect both DNA and histone methylation [36].

We found that females who in the reduced folate carrier gene, *SLC19A1*, had A allele variant at the nucleotide position c.80 showed increased lung cancer risk. To our knowledge, this is the first study to report this association. Sex-specific concordance in relation to lung cancer risk might be explained by differences in the activity of oestrogen receptors which were reported in lung adenocarcinoma cells derived from subjects of different gender [37], while the results from the studies of non-small cell lung cancer suggest that these tumours may produce oestradiol which can stimulate the growth of oestrogen receptor-positive malignant cells [38]. Moreover, sex-specific determinants of folate/homocysteine pathway have already been indicated [39]. Interestingly, it has been reported previously that young women, but not men, who carried the A allele, had higher RBC folate concentrations [40]. In this study we report a trend towards an increased lung cancer risk among subjects in the upper half of RBC folate distribution and an increased risk for those in the upper half of serum folate distribution. By linking the published results and our findings we could suspect that female carriers of the *SLC19A1* c80AA genotype might have had higher levels of folates during their lifetime as compared to others. This, in combination with smoking habit might result in increased lung cancer risk.

The most widely studied is the *MTHFR* c.665C>T polymorphism, yet its role in the aetiology of cancer remains controversial. The *MTHFR* c.665TT genotype, encoding the thermolabile enzyme with a lower affinity for its cofactor, flavin adenine dinucleotide (FAD), has been associated with an increased risk of lung cancer in populations analysed as a whole [18–20, 22] and in the subgroups: of patients with earlier onset or low folate intake [16], in women [17] and in squamous and small cell lung carcinomas [15]. However, the TT genotype has also been associated with a decreased risk of lung cancer [21] and no association between the *MTHFR* c.665C>T genotypes and lung cancer risk has been found [11–14]. Our results present a trend towards a protective effect of the *MTHFR* c.665T allele on lung cancer risk.

Similarly, the *MTHFR* c.1286CC genotype has been associated with an increased lung cancer risk in the general population [17, 20] and among non-smokers [23], with a reduced risk [16] or no risk [11, 14, 15, 18, 19, 22]. In our population, no association between this polymorphism and lung cancer risk was found.

The *TYMS* 6 bp ins allele within the 3’UTR has been reported to contribute to a higher risk of lung cancer [25] and we also observed a similar trend. As in the previous studies, we found no association between the *MTR* c.2756A>G [14–16, 18], *MTRR* c.66A [14, 19], *SHMT1* c.1420C>T [14, 24] and *TYMS*: 5’UTR 2R/3R [15] polymorphisms and lung cancer risk in a general population. However, in a study comprising 2183 subjects, the combined *MTR* c.2756G and *MTRR* c.66A alleles have been linked to a higher risk [17].

Several polymorphisms in the C1 metabolism genes were associated with altered folate/homocysteine levels. Importantly, the association between the polymorphisms and folate/homocysteine status may be suppressed by high folate levels. The impact of the C1 polymorphisms on lung cancer risk, if observed, is most likely due to altering either folate levels or distribution of folate forms. Therefore, it is worth to interpret the effect the polymorphisms in the C1 metabolism genes might have on lung cancer outcome in the context of folate profiles for evaluated populations, as reported in this study.

Food fortification with folic acid has been introduced in several countries. However, this practice has been criticised due to its potentially dangerous effects. Our results suggest that high folate levels may be associated with an increased risk of lung cancer in cigarette smokers, a finding that may support concerns of food fortification with folic acid.

While designing the study, several steps were taken to reduce the risk of false discovery. The strength of the study is the rigorous matching of cases and controls, and that study subjects inhabit a relatively confined area in northern Poland. However, several potential confounders should be listed that might have biased our findings. Even though all subjects were diagnosed with LDCT, preclinical disease in control subjects cannot be excluded. The important limitation of the study is the lack of cotinine measurements (smoking status and pack-years of smoking were self-reported). There are several factors that are known to correlate with B-vitamins status on which information was not available such as educational level or lifestyle e.g. physical activity or dietary habits. Further, the disease might have been induced by undisclosed occupational exposures, which might have been incidentally correlated to higher folate measures in lung cancer cases. In addition, the majority of blood draws were from non-fasting subjects.

## Conclusions

**O**ur results suggest that serum folate levels in the upper half of the distribution are associated with a moderately increased lung cancer risk among smokers from northern Poland. If so, relatively small differences in folate concentrations may support growth and progression of smoking induced lung cancer. We believe it would be interesting to repeat this study in other populations of smokers, with a strict matching of cases and controls for pack-years of cigarettes smoked, to test if the observed effect is general. Further, the female carriers of the *SLC19A1* c.80A allele belong to the group exhibiting increased lung cancer incidence pointing that certain associations between the folate/homocysteine pathway and the development of smoking-related lung cancer are sex-specific.

## Supporting information

S1 Fig Correlations between the metabolite values determined from differently treated aliquots of randomly selected samples. A) folate concentration determined from serum samples within a day of blood collection vs. serum samples frozen for a few days; B) folate concentrations determined from serum samples frozen for 34–48 days vs. serum samples frozen for 10 months; C) homocysteine concentrations determined from serum samples frozen for 34–48 days vs. serum samples frozen for 10 months; D) RBC folate concentrations determined from whole blood vs. whole blood diluted 1:10 in 1% ascorbic acid, frozen for 2 years. The measures were corrected for the hematocrit values. Correlations were calculated using Pearson statistics.
(PDF)

S1 Table Genotyping conditions. (PDF)

S2 Table Median (interquartile ranges), N, of serum folate, RBC folate, corrected RBC folate and homocysteine defined by genotypes in C1 metabolism genes. (PDF)

S3 Table Median (interquartile ranges), N, of serum folate, RBC folate, corrected RBC folate and homocysteine defined by the *SLC19A1* c.80G>A genotypes and sex. (PDF)

S1 Dataset Raw data file. (XLS)
