# Supplementary material for: Folate/homocysteine metabolism and lung cancer risk among smokers
Source: PLoS One. 2019 Apr 2;14(4):e0214462. doi: 10.1371/journal.pone.0214462 (PMC6445430; doi:10.1371/journal.pone.0214462)
Supplement: S1 Fig — A) folate concentration determined from serum samples within a day of blood collection vs. serum samples frozen for a few days; B) folate concentrations determined from serum samples frozen for 34–48 days vs. serum samples frozen for 10 months; C) homocysteine concentrations determined from serum samples frozen for 34–48 days vs. serum samples frozen for 10 months; D) RBC folate concentrations determined from whole blood vs. whole blood diluted 1:10 in 1% ascorbic acid, frozen for 2 years. The measures were corrected for the hematocrit values. Correlations were calculated using Pearson statistics. (PDF) [file pone.0214462.s001.pdf]

Folate/homocysteine metabolism and lung cancer risk among smokers

Stanisławska-Sachadyn A, Borzyszkowska J, Krzemiński M, Janowicz A, Dziadziuszko R, Jassem J, Rzyman W, Limon J

Supporting information, S1 Fig

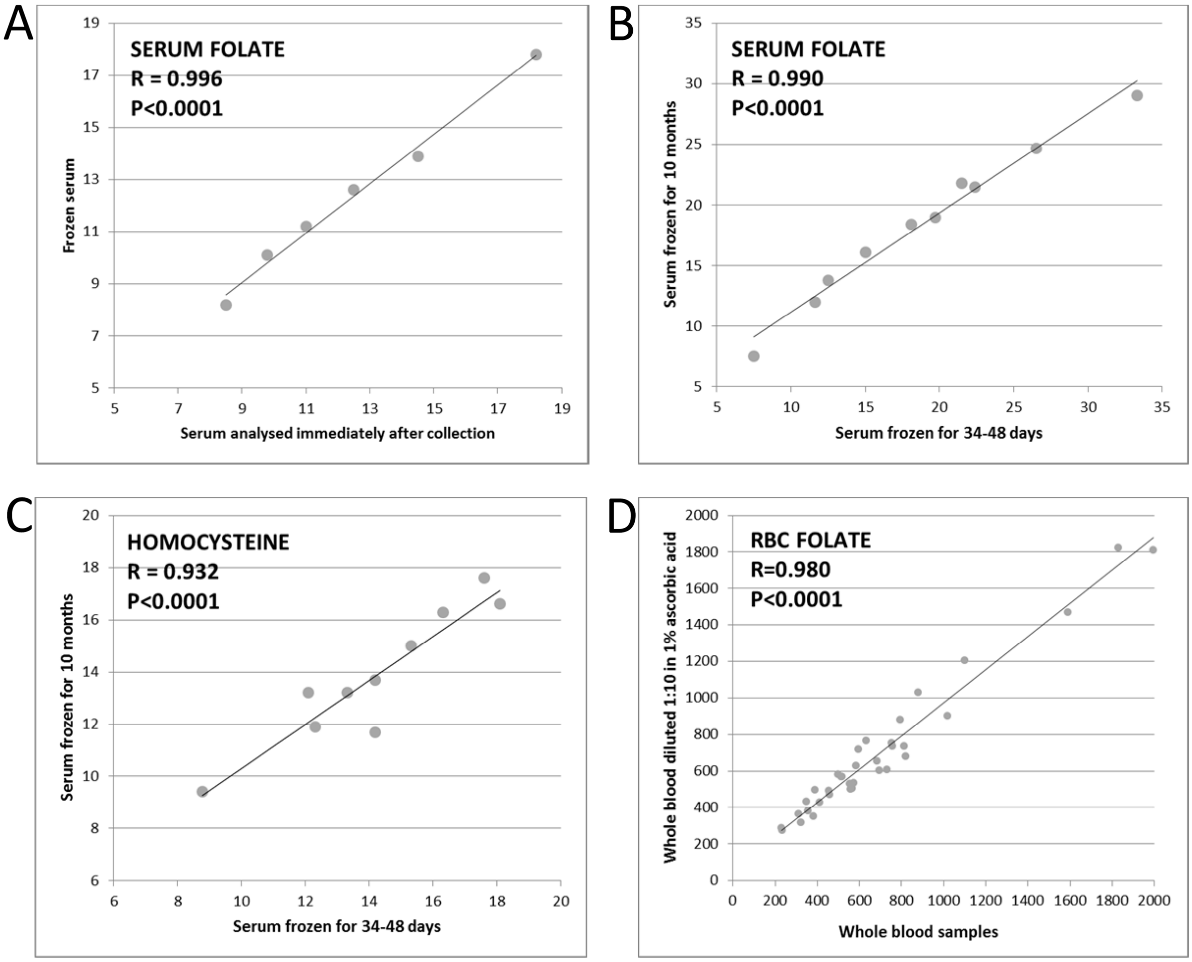

S1 Fig. Correlations between the metabolite values determined from differently treated aliquots of randomly selected samples A) folate concentration determined from serum samples within a day of blood collection vs. serum samples frozen for a few days; B) folate concentrations determined from serum samples frozen for 34-48 days vs. serum samples frozen for 10 months; C) homocysteine concentrations determined from serum samples frozen for 34-48 days vs. serum samples frozen for 10 months; D) RBC folate concentrations determined from whole blood vs. whole blood diluted 1:10 in 1% ascorbic acid, frozen for 2 years. The measures were corrected for the hematocrit values.

Correlations were calculated using Pearson statistics.
