# Supplementary material for: Folate/homocysteine metabolism and lung cancer risk among smokers
Source: PLoS One. 2019 Apr 2;14(4):e0214462. doi: 10.1371/journal.pone.0214462 (PMC6445430; doi:10.1371/journal.pone.0214462)
Supplement: S1 Table — (PDF) [file pone.0214462.s002.pdf]

Folate/homocysteine metabolism and lung cancer risk among smokers

Stanisławska-Sachadyn A, Borzyszkowska J, Krzemiński M, Janowicz A, Dziadziuszko R, Jassem J, Rzyman W, Limon J

Supporting information, S1 Table

S1 Table. Genotyping conditions

Polymorphisms	Probes, primers, other reagents		Thermal profile
**Taqman method**			
rs1801133	T: 6FAM-ATGAAATCGACTCCCGC-MGBNFQ*	50 nM	95ºC for 10 min
MTHFR c.665C>T	C: VIC-ATGAAATCGGCTCCCGC-MGBNFQ*	50 nM	95ºC for 30 s
	F: GCAGGGAGCTTTGAGGCTGACC [1]	0.5 μM	59ºC for 1 min
	R: TGGGGCAAGTGATGCCCATGT [1]	0.5 μM	repeat 50 times
rs1801131	C: 6FAM-AGACACTTGCTTCACT-MGBNFQ*	50 nM	95ºC for 10 min
MTHFR c.1286A>C	A: VIC-CAAAGACACTTTCTTC-MGBNFQ*	50 nM	95ºC for 30 s
	F: GAGGAGCTGCTG AAGATG T [1]	0.5 μM	56ºC for 1 min
	R: CGAGAGGTAAAGAACGAAGA [1]	0.5 μM	repeat 50 times
rs1805087	G: 6FAM-ACAGGGCCATTATG-MGBNFQ*	50 nM	95ºC-10 min
MTR c.2756A>G	A: VIC-ATTAGACAGGACCATTATG-MGBNFQ*	50 nM	95 ºC -30 s
	F: AGTGTTCCCAGCTGTTAGATGA [1]	0.5 μM	62 ºC -1 min
	R: TGTTTCTACCACTTA CCTTGAGAGACT*	0.5 μM	repeat 50 times
rs1801394	G: 6FAM-CTTGCTCACACATTT-MGBNFQ*	50 nM	95ºC-10 min
MTRR c.66A>G	A: VIC-TGCTCACATATTTC-MGBNFQ*	50 nM	92 ºC -30 s
	F: CATGCCTTGAAGTGATGAGG*	0.5 μM	60 ºC -1 min
	R: GATCTGCAGAAAATCCATGTACCA*	0.5 μM	repeat 50 times
rs1979277	C: 6FAM-CGCCTCTCTCTTC-MGBNFQ [2]	50 nM	95ºC-10 min
SHMT1 c.1420C>T	T: VIC-TCGCCTCTTTCTTC-MGBNFQ [3]	50 nM	95 ºC -30 s
	F: AGGAGAGACTGGCAGGGGAT [3]	0.5 μM	60 ºC -60 s
	R: CATCCATCTCTCAGGTGGGG [3]	0.5 μM	repeat 50 times
rs61510559	T: 6FAM-CGAGGTGCCGCCAG-MGBNFQ [4]	100 nM	95ºC-10 min
SLC19A1 c.80G>A	C: VIC-CACGAGGCGCCGC-MGBNFQ [4]	50 nM	95 ºC -30 s
	F: GGCCTGACCCCGAGCT [4]	0.5 μM	63 ºC -1 min
	R: AGCCGTAGAAGCAAAGGTAGCA [4]	0.5 μM	repeat 60 times
rs2236225	G: 6FAM-TGCAGACCGGATC-MGBNFQ [3]	50 nM	95ºC-10 min
MTHFD1 c.1958G>A	A: VIC-AGACCAGATCGCACT-MGBNFQ [3]	50 nM	95 ºC -30 s
	F: GTTTGCCAACATCGCACAT [3]	0.5 μM	60 ºC -1 min
	R: CATACAGTCTATTCACTGGTTTTGC [3]	0.5 μM	repeat 50 times

rs1801198	G: 6FAM-CCCCACGCATGGG–MGBNFQ*	50 nM	96ºC 5 min
TCN2 c.776C>G	C: VIC-CCCCAGGCATGGG-MGBNFQ*	100 nM	96ºC 10 s
	F: TCACCAGTTCCTCATGACTT	0.5 µM	60ºC 10 s
	R: GAGCATTCTGGAAGGCT	0.5 µM	72ºC 20 s
			repeat 50 times
rs202243265	ins: 6FAM-TGGTTATGAACTTTATAGTTG-MGBNFQ [4]	50 nM	95ºC-10 min
rs11280056	del: VIC-TGGTTATGAACTTTAAAGTT-MGBNFQ [4]	50 nM	92 ºC -30 s
rs16430	F: AGCTGAGTAACACCATCGATCATG [4]	0.5 µM	60 ºC -1 min
TYMS 3'UTR ins/del	R: GGACGAATGCAGAACACTTCTTTA [4]	0.5 µM	repeat 50 times
**PCR method**			
TYMS 5'UTR 2R/3R	F: GTGGCTCCTGCGTTTCCCCC [5]	0.5 µM	96ºC-5 min
	R: CCGCGCCATGCCTGTGGCCGGCTCGGAGC	0.5 µM	96 ºC -1 min
	dNTPs	800 µM	64 ºC -1 min
	10x conc. PCR buffer (Thermo Scientific)	2 µl	72 ºC -1 min
	$MgCl_2$	1.5 mM	repeat 35 times
	Maxima Hot Start Taq DNA polymerase (Thermo Scientific)	0.35 U	72 ºC -5 min
	DMSO	0.5 µl	
	Genomic DNA	2 µl	
	Total volume	20 µl	
DHFR	F :CCACGGTCGGGGTACCTGGG [6]	0.5 µM	94ºC-4 min
c.86+60_78ins22	F: ACGGTCGGGGTGGCCGACTC [6]	0.5 µM	94 ºC -55 s
	R: AAAAGGGGAATCCAGTCGG [6]	1 µM	65 ºC - 55 s
	dNTPs	800 µM	72 ºC - 55 s
	10x conc. PCR buffer (Thermo Scientific)	2.5 µl	repeat 40 times
	$MgCl_2$	2.5 mM	72 ºC -12 min
	Taq DNA polymerase (Thermo Scientific)	0.25 U	
	Genomic DNA	5 µl	
	Total volume	25 µl	
CBS c.844_845ins68	F: TATTGGCCACTCCCATAATAGA [7]	0.5 µM	94ºC-5 min
	R: CGGCTCTGCGAGGATGGACCCTT [7]	0.5 µM	94 ºC -1 min
	dNTPs	800 µM	59 ºC -1 min
	10x conc. PCR buffer (Thermo Scientific)	2 µl	72 ºC -1 min
	$MgCl_2$	1.5 mM	repeat 35 times
	Taq DNA polymerase (Thermo Scientific)	0.25 U	72 ºC -5 min
	Genomic DNA	2 µl	
	Total volume	20 µl	

*primers / probes sequences were obtained from SNP500 Cancer website (http://snp500cancer.nci.nih.gov/cgfseq/pages/snp500.do).

References

1. Summers CM, Cucchiara AJ, Nackos E, Hammons AL, Mohr E, Whitehead AS, et al. Functional polymorphisms of folate-metabolizing enzymes in relation to homocysteine concentrations in systemic lupus erythematosus. J Rheumatol. 2008;35(11):2179-86.
2. Skibola CF, Smith MT, Kane E, Roman E, Rollinson S, Cartwright RA, et al. Polymorphisms in the methylenetetrahydrofolate reductase gene are associated with susceptibility to acute leukemia in adults. Proc Natl Acad Sci U S A. 1999;96(22):12810-5.
3. Summers CM, Mitchell LE, Stanislawska-Sachadyn A, Baido SF, Blair IA, Von Feldt JM, et al. Genetic and lifestyle variables associated with homocysteine concentrations and the distribution of folate derivatives in healthy premenopausal women. Birth Defects Res A Clin Mol Teratol. 2010;88(8):679-88.
4. Skibola CF, Forrest MS, Coppede F, Agana L, Hubbard A, Smith MT, et al. Polymorphisms and haplotypes in folate-metabolizing genes and risk of non-Hodgkin lymphoma. Blood. 2004;104(7):2155-62.
5. Brown KS, Kluijtmans LA, Young IS, McNulty H, Mitchell LE, Yarnell JW, et al. The thymidylate synthase tandem repeat polymorphism is not associated with homocysteine concentrations in healthy young subjects. Hum Genet. 2004;114(2):182-5.
6. Johnson WG, Stenroos ES, Spychala JR, Chatkupt S, Ming SX, Buyske S. New 19 bp deletion polymorphism in intron-1 of dihydrofolate reductase (DHFR): a risk factor for spina bifida acting in mothers during pregnancy? Am J Med Genet A. 2004;124A(4):339-45.
7. Barbaux S, Kluijtmans LA, Whitehead AS. Accurate and rapid "multiplex heteroduplexing" method for genotyping key enzymes involved in folate/homocysteine metabolism. Clin Chem. 2000;46(7):907-12.
