# Supplementary material for: Folate/homocysteine metabolism and lung cancer risk among smokers
Source: PLoS One. 2019 Apr 2;14(4):e0214462. doi: 10.1371/journal.pone.0214462 (PMC6445430; doi:10.1371/journal.pone.0214462)
Supplement: S2 Table — (PDF) [file pone.0214462.s003.pdf]

Folate/homocysteine metabolism and lung cancer risk among smokers

Stanisławska-Sachadyn A, Borzyszkowska J, Krzemiński M, Janowicz A, Dziadziuszko R, Jassem J, Rzyman W, Limon J

Supporting information, S2 Table

S2 Table. Median (interquartile ranges), N, of serum folate, RBC folate, corrected RBC folate and homocysteine defined by genotypes in C1 metabolism genes.
Two outlying extreme values were deleted from the analyses: serum folate = 800.5; 565.4 nmol/l.

Variable		Lung cancer cases*	Controls*	$P_{wilcoxon}$
	MTHFR c.665C>T			
Serum folates nmol/L	CC	18.8 (14.3-24.7), 65	17.7 (13.4-23.4), 157	0.383
	CT	20.4 (13.6-24.0), 55	17.6 (12.9-22.7), 198	0.227
	TT	16.75 (10.3-20.6), 12	16.1 (11.1-22.0), 39	0.885
	$P_{Kruskal-Wallis}$	0.505	0.591	
RBC folates nmol/L	CC	533.3 (436.1-721.9), 47	508.35 (363.3-697.9), 110	0.538
	CT	563.3 (378.75-674.8), 36	496.4 (382.7-638.1), 147	0.463
	TT	627.35 (349.6-972.1), 10	607.1 (482.6-852.2), 22	0.889
	$P_{Kruskal-Wallis}$	0.714	0.108	
RBC folates corrected nmol/L	CC	512.6 (401.4-688.0), 47	473.05 (345.6-680.0), 110	0.582
	CT	527.9 (358.5-634.8), 36	470.95 (362.3-602.0), 146	0.454
	TT	609.55 (335.2-942.0), 10	594.95 (457.7-827.7), 22	0.889
	$P_{Kruskal-Wallis}$	0.624	0.082	
Homocysteine µmol/L	CC	15.2 (11.4-18.6), 63	14.4 (11.6-18.5), 157	0.954
	CT	15.55 (11.4-18.3), 54	14.8 (12.2-18.7), 199	0.488
	TT	16.75 (14.85-20.5), 12	15.3 (12.3-18.9), 39	0.297
	$P_{Kruskal-Wallis}$	0.365	0.838	
	MTHFR c.1268A>C			
Serum folates nmol/L	AA	19.7 (14.3-23.8), 69	17.7 (13.25-23.9), 192	0.606
	AC	18.1 (14.3-25.4), 49	16.5 (12.7-22.4), 169	0.174
	CC	19.15 (14.1-28.8), 14	19.9 (12.5-23.4), 33	0.816
	$P_{Kruskal-Wallis}$	0.935	0.475	
RBC folates nmol/L	AA	539.5 (372.3-756.7), 51	516.75 (388.4-670.4), 138	0.487
	AC	560.4 (411.6-661.4), 31	481.1 (358.5-642.2), 115	0.345
	CC	566.4 (442-791.3), 11	672.2 (468.9-831.1), 26	0.332
	$P_{Kruskal-Wallis}$	0.746	0.006	
RBC folates corrected nmol/L	AA	515.2 (343.5-729.2), 51	477.6 (367.7-633.3), 137	0.480
	AC	531.1 (378.6-622.8), 31	446.6 (345.5-601.8), 115	0.373
	CC	541.8 (407.6-764.1), 11	642.3 (451.8-794.8), 26	0.349
	$P_{Kruskal-Wallis}$	0.762	0.005	
Homocysteine µmol/L	AA	15.88 (11.7-19.6), 67	15.0 (12.0-18.8), 193	0.602
	AC	15.4 (11.2-17.25), 48	14.6 (12.1-18.75), 168	0.328
	CC	14.85 (11.3-18.6), 14	14.05 (11.3-16.0), 34	0.602
	$P_{Kruskal-Wallis}$	0.443	0.187	
	CBS c.844_845ins68			
Serum folates nmol/L	DD	19.15 (14.3-24.3), 110	17.5 (12.9-23.1), 341	0.248
	I allele	19.85 (13.8-25.6), 22	17.0 (14.5-22.4), 53	0.503
	$P_{Kruskal-Wallis}$	0.710	0.904	
RBC folates nmol/L	DD	554.0 (395.7-711.25), 80	506.5 (383.3-677.4), 249	0.512
	I allele	560.4 (457.6-773.1), 13	508.7 (398.9-660.4), 30	0.488
	$P_{Kruskal-Wallis}$	0.603	0.979	

RBC folates corrected nmol/L	DD	520.55 (370.2-677.1), 80	474.6 (364.65-645.8), 248	0.544
	I allele	534.7 (441.5-731.7), 13	478.5 (376.3-633.3), 30	0.456
	$P_{Kruskal-Wallis}$	0.580	0.977	
Homocysteine µmol/L	DD	16.0 (11.8-18.7), 107	14.8 (12.1-18.7), 342	0.971
	I allele	14.5 (10.2-18.3), 22	12.8 (11.7-17.7), 53	0.991
	$P_{Kruskal-Wallis}$	0.338	0.057	
	SHMT1 c.1420C>T			
Serum folates nmol/L	CC	20.4 (13.6-24.3), 53	17.2 (12.2-22.4), 164	0.203
	CT	18.6 (14.3-24.5), 61	17.7 (13.8-23.1), 185	0.649
	TT	20.05 (15.6-21.5), 18	15.6 (12.2-24.3), 45	0.589
	$P_{Kruskal-Wallis}$	0.966	0.499	
RBC folates nmol/L	CC	566.2 (372.3-733.7), 37	483.3 (383.5-643.3), 119	0.217
	CT	560.4 (401.5-675.2), 49	532.2 (387.25-741.55), 124	0.721
	TT	533.3 (443.4-790.9), 7	511.95 (356.05-669.6), 36	0.551
	$P_{Kruskal-Wallis}$	0.859	0.349	
RBC folates corrected nmol/L	CC	524.7 (343.5-705.9), 37	458.4 (367.7-624.6), 119	0.261
	CT	531.1 (378.4-653.6), 49	507.9 (370.5-699.6), 123	0.727
	TT	512.6 (415.1-752.0), 7	473.8 (336.4-635.05), 36	0.529
	$P_{Kruskal-Wallis}$	0.827	0.403	
Homocysteine µmol/L	CC	14.2 (10.3-17.8), 53	14.7 (11.8-18.65), 164	0.263
	CT	16.6 (13.6-18.7), 58	14.7 (12.2-18.3), 185	0.188
	TT	14.95 (11.2-19.6), 18	14.65 (12.4-18.9), 46	0.565
	$P_{Kruskal-Wallis}$	14.2 (10.3-17.8), 53	0.902	
	MTHFD1 c.1958G>A			
Serum folates nmol/L	GG	19.6 (14.5-24.3), 30	18.9 (13.1-23.8), 82	0.679
	GA	18.4 (13.6-24.7), 63	17.5 (12.9-22.9), 182	0.657
	AA	20.4 (14.3-24.7), 39	16.65 (12.0-22.4), 130	0.195
	$P_{Kruskal-Wallis}$	0.790	0.439	
RBC folates nmol/L	GG	582.3 (507.05-808.0), 24	524.4 (391.2-741.4), 59	0.136
	GA	522.35 (362.85-712.65), 44	508.35 (382.65-636.65), 124	0.533
	AA	523.6 (371.8-656.0), 25	497.6 (383.0-728.35), 96	0.638
	$P_{Kruskal-Wallis}$	0.205	0.624	
RBC folates corrected nmol/L	GG	556.75 (475.3-776.75), 24	486.1 (370.5-701.2), 59	0.141
	GA	502.5 (340.95-677.1), 44	476.05 (364.3-599.55), 124	0.560
	AA	501.6 (328.5-616.3), 25	470.9 (362.3-707.6), 95	0.610
	$P_{Kruskal-Wallis}$	0.204	0.648	
Homocysteine µmol/L	GG	16.6 (11.2-18.7), 29	14.7 (12.5-19.6), 83	0.974
	GA	15.8 (11.7-18.6), 61	14.9 (12.0-18.7), 181	0.881
	AA	14.8 (11.4-18.1), 39	14.4 (11.6-18.0), 131	0.979
	$P_{Kruskal-Wallis}$	0.746	0.504	
	MTRR c.66A>G			
Serum folates nmol/L	GG	20.4 (15.9-24.3), 50	18.1 (12.7-23.1), 119	0.154
	GA	18.8 (13.6-22.9), 58	17.6 (13.1-23.4), 190	0.792
	AA	19.6 (13.6-26.55), 24	16.3 (12.5-22.0), 85	0.226
	$P_{Kruskal-Wallis}$	0.337	0.593	
RBC folates nmol/L	GG	533.3 (372.3-702.6), 35	462 (333.8-655.1), 81	0.314
	GA	579.5 (442.0-733.7), 39	513.6 (421-667.0), 134	0.383
	AA	527.5 (371.8-755.0), 19	559.4 (386.65-736.45), 64	0.884
	$P_{Kruskal-Wallis}$	0.707	0.205	

RBC folates corrected nmol/L	GG	508.8 (343.5-676.9), 35	441.0 (319.4-621.9), 81	0.304
	GA	565.5 (405.7-692.7), 39	482.3 (389.0-636.6), 134	0.438
	AA	515.2 (324.7-722.2), 19	527.3 (362.3-699.1), 63	0.965
	$P_{Kruskal-Wallis}$	0.694	0.194	
Homocysteine µmol/L	GG	15.4 (11.9-19.65), 48	14.9 (11.8-18.7), 118	0.909
	GA	15.6 (11.4-18.3), 57	14.7 (12.2-18.7), 190	0.618
	AA	16.2 (10.55-18.75), 24	14.2 (12.0-17.7), 87	0.821
	$P_{Kruskal-Wallis}$	0.870	0.822	
	MTR c.2756A>G			
Serum folates nmol/L	AA	19.0 (14.1-23.6), 91	18.1 (13.4-23.1), 243	0.765
	AG	19.85 (14.3-25.85), 36	16.5 (12.0-23.1), 133	0.154
	GG	24.0 (14.7-25.6), 5	14.95 (12.2-20.4), 18	0.526
	$P_{Kruskal-Wallis}$	0.669	0.253	
RBC folates nmol/L	AA	539.5 (389.9-675.2), 65	505.6 (388.4-672.2), 174	0.482
	AG	566.2 (428.5-719.9), 25	517.4 (375.4-725.9), 93	0.730
	GG	745.8 (354.9-755), 3	475.5 (374.5-588.2), 12	0.536
	$P_{Kruskal-Wallis}$	0.811	0.822	
RBC folates corrected nmol/L	AA	515.2 (362-653.6), 65	474.4 (370.8-633.5), 173	0.453
	AG	534.7 (389.3-684.4), 25	497.5 (359.8-699.6), 93	0.782
	GG	688.0 (314.5-722.2), 3	434.55 (360.6-566.65), 12	0.633
	$P_{Kruskal-Wallis}$	0.891	0.754	
Homocysteine µmol/L	AA	15.7 (11.2-18.3), 89	14.7 (12-18.6), 245	0.538
	AG	15.88 (12.5-19.2), 35	14.5 (11.85-18.5), 132	0.426
	GG	14.8 (13.7-19.8), 5	16.45 (12.5-18.7), 18	0.857
	$P_{Kruskal-Wallis}$	0.769	0.655	
	TYMS 3'UTR ins/del			
Serum folates nmol/L	II	19.7 (13.6-24), 67	18.1 (13.8-23.4), 179	0.789
	ID	18.1 (14.3-24.3), 61	17.1 (12.9-23.1), 182	0.345
	DD	22.45 (17.55-25.2), 4	13.8 (11.1-17.7), 33	0.078
	$P_{Kruskal-Wallis}$	0.744	0.032	
RBC folates nmol/L	II	602.0 (444.2-756.7), 46	502.05 (372.3-727.5), 126	0.111
	ID	491.55 (372.05-632.5), 44	515.4 (391.6-670.0), 125	0.441
	DD	411.6 (259.8-1446.6), 3	454.2 (364.55-580.45), 28	0.875
	$P_{Kruskal-Wallis}$	0.132	0.372	
RBC folates corrected nmol/L	II	572.4 (405.7-729.2), 46	473.45 (352.3-680.0), 126	0.115
	ID	468.85 (340.95-605.5), 44	486.55 (372.15-634.5), 124	0.456
	DD	378.6 (242.2-1406.7), 3	425.8 (340.35-560.25), 28	0.875
	$P_{Kruskal-Wallis}$	0.124	0.428	
Homocysteine µmol/L	II	14.8 (11.4-17.5), 65	15.5 (12.7-19.2), 179	0.050
	ID	16.25 (11.95-19.5), 60	13.6 (11.6-18.3), 183	0.173
	DD	18.65 (17.55-20.05), 4	14.7 (12.0-17.7), 33	0.056
	$P_{Kruskal-Wallis}$	0.060	0.042	
	TYMS 5'UTR 2R/3R			
Serum folates nmol/L	2R/2R	20.5 (15.15-25.15), 32	18.5 (14.4-22.65), 72	0.317
	2R/3R	18.6 (13.8-24.3), 66	17.0 (12.5-23.1), 205	0.504
	3R/3R	19.05 (14.5-24.0), 34	17.2 (12.7-22.9), 117	0.477
	$P_{Kruskal-Wallis}$	0.522	0.400	
RBC folates nmol/L	2R/2R	570.4 (395.5-748.75), 20	512.6 (385.3-684.2), 47	0.709
	2R/3R	567.0 (411.6-733.7), 45	541.5 (391.6-689.2), 146	0.526
	3R/3R	521.85 (351.55-671.7), 28	454.55 (358.5-619.7), 86	0.538
	$P_{Kruskal-Wallis}$	0.412	0.069	

RBC folates corrected nmol/L	2R/2R	535.8 (368.3-712.2), 20	477.0 (367.0-657.7), 47	0.737
	2R/3R	541.8 (382.4-688.0), 45	517.85 (371.5-658.7), 146	0.548
	3R/3R	494.65 (326.6-628.8), 28	430.1 (343.6-578.4), 85	0.552
	$P_{Kruskal-Wallis}$	0.396	0.049	
Homocysteine µmol/L	2R/2R	14.8 (11.2-18.1), 31	15.95 (12.15-20.2), 72	0.222
	2R/3R	15.55 (11.95-18.7), 64	14.3 (11.75-18.15), 204	0.415
	3R/3R	16.3 (11.4-19.4), 34	14.7 (12.1-18.8), 119	0.949
	$P_{Kruskal-Wallis}$	0.705	0.103	
	DHFR c.86+60_78ins22			
Serum folates nmol/L	II	18.5 (13.95-22.9), 36	17.7 (13.1-23.6), 119	0.909
	ID	20.4 (14.4-25.6), 72	17.2 (12.6-22.9), 196	0.042
	DD	18.0 (12.1-23.45), 24	17.5 (12.7-22.7), 79	0.827
	$P_{Kruskal-Wallis}$	0.222	0.747	
RBC folates nmol/L	II	466.5 (353.2-656.0), 31	512.8 (429.3-670.0), 86	0.194
	ID	590.9 (457.25-766.15), 48	506.3 (354.9-688.85), 136	0.068
	DD	544.9 (372.3-682.0), 14	492.2 (382.7-616.7), 57	0.823
	$P_{Kruskal-Wallis}$	0.055	0.661	
RBC folates corrected nmol/L	II	441.5 (328.5-622.8), 31	486.55 (398.3-628.1), 86	0.171
	ID	563.5 (439.55-729.1), 48	469.1 (332.9-654.5), 135	0.065
	DD	513.15 (343.5-641.3), 14	464.0 (361.4-595.0), 57	0.868
	$P_{Kruskal-Wallis}$	0.066	0.633	
Homocysteine µmol/L	II	16.4 (11.9-17.8), 35	14.6 (12.0-18.5), 118	0.434
	ID	14.7 (11.2-18.3), 70	14.5 (12.4-18.7), 198	0.366
	DD	15.84 (12.3-19.9), 24	15.0 (11.7-18.6), 79	0.728
	$P_{Kruskal-Wallis}$	0.503	0.907	
	TCN2 c.776C>G			
Serum folates nmol/L	CC	19.3 (13.8-23.8), 39	17.0 (12.7-23.1), 124	0.450
	CG	19.5 (14.75-24.7), 64	17.8 (12.9-23.4), 182	0.273
	GG	18.1 (13.6-24.0), 29	17.35 (13.4-22.1), 88	0.820
	$P_{Kruskal-Wallis}$	0.638	0.829	
RBC folates nmol/L	CC	623.35 (446.5-692.3), 24	513.7 (381.8-670.4), 89	0.188
	CG	548.3 (419.5-752.4), 45	494.5 (392.3-684.7), 129	0.470
	GG	491.55 (339.7-722.3), 24	510.2 (346.4-652.1), 61	0.643
	$P_{Kruskal-Wallis}$	0.369	0.972	
RBC folates corrected nmol/L	CC	596.15 (417.95-665.25), 24	478.5 (359.1-642.5), 89	0.192
	CG	516.4 (382.4-729.2), 45	460.2 (370.75-643.25), 128	0.510
	GG	468.85 (309.9-685.0), 24	472.5 (332.2-614.7), 61	0.588
	$P_{Kruskal-Wallis}$	0.342	0.955	
Homocysteine µmol/L	CC	13.75 (10.2-16.7), 38	14.2 (12.0-17.7), 125	0.093
	CG	16.5 (12-18.8), 62	14.7 (11.7-18.9), 182	0.516
	GG	16.4 (13.7-20.6), 29	15.45 (13.1-19.45), 88	0.611
	$P_{Kruskal-Wallis}$	0.014	0.153	
	SLC19A1 c.80G>A			
Serum folates nmol/L	GG	18.1 (12.7-23.8), 35	16.4 (12.9-23.1), 114	0.941
	GA	19.15 (14.4-23.8), 68	17.7 (12.7-23.4), 190	0.483
	AA	22.4 (15.6-25.4), 29	17.7 (13.4-22.2), 90	0.035
	$P_{Kruskal-Wallis}$	0.194	0.933	
RBC folates nmol/L	GG	572.4 (434-733.25), 28	511.85 (388.2-755.45), 80	0.512
	GA	546.85 (396.25-692.3), 44	515.2 (387.05-688.45), 140	0.924
	AA	517.2 (371.8-756.7), 21	482.6 (372.3-639.7), 59	0.330
	$P_{Kruskal-Wallis}$	0.726	0.331	

RBC folates corrected nmol/L	GG	539.3 (409.95-708.85), 28	484.2 (371.5-705.6), 79	0.485
	GA	519.95 (370.3-659.1), 44	488.4 (367.35-656.6), 140	0.970
	AA	481.9 (332.9-726.5), 21	452.9 (347.5-621.5), 59	0.370
	$P_{Kruskal\text{-}Wallis}$	0.739	0.320	
Homocysteine µmol/L	GG	15.25 (12.0-18.3), 34	14.45 (12.0-18.7), 114	0.897
	GA	16.3 (11.2-18.7), 67	14.7 (11.9-18.4), 191	0.925
	AA	15.45 (11.85-16.9), 28	15.55 (12.5-18.9), 90	0.688
	$P_{Kruskal\text{-}Wallis}$	0.957	0.857	
