# Supplementary material for: Folate/homocysteine metabolism and lung cancer risk among smokers
Source: PLoS One. 2019 Apr 2;14(4):e0214462. doi: 10.1371/journal.pone.0214462 (PMC6445430; doi:10.1371/journal.pone.0214462)
Supplement: S3 Table — (PDF) [file pone.0214462.s004.pdf]

Folate/homocysteine metabolism and lung cancer risk among smokers

Stanisławska-Sachadyn A, Borzyszkowska J, Krzemiński M, Janowicz A, Dziadziuszko R, Jassem J, Rzyman W, Limon J

Supporting information, S3 Table

S3 Table. Median (interquartile ranges), N, of serum folate, RBC folate, corrected RBC folate and homocysteine defined by the *SLC19A1* c.80G>A genotypes and sex.

Two outlying extreme values were deleted from the analyses: serum folate = 800.5; 565.4 nmol/l.

Variable	SLC19A1	Lung cancer cases*	Controls*	$P_{wilcoxon}$
	Females			
Serum folates nmol/L	GG	18.6 (13.1-24), 11	17.7 (14.5-27.4), 57	0.868
	GA	19.3 (15.4-25.6), 31	18.1 (13.1-24.3), 94	0.377
	AA	22.9 (14.3-25.4), 19	17.9 (12.9-22.9), 31	0.139
	$P_{Kruskal-Wallis}$	0.687	0.643	
RBC folates nmol/L	GG	525.8 (348.8-768.1), 8	520.5 (425-697.9), 39	0.857
	GA	502.3 (349.25-692.3), 20	525.8 (381.8-725.9), 66	0.606
	AA	517.2 (419.5-721.9), 13	441.3 (375.4-677.4), 18	0.650
	$P_{Kruskal-Wallis}$	0.876	0.586	
RBC folates corrected nmol/L	GG	494.75 (318.1-738.95), 8	489.3 (412.4-682.4), 39	0.813
	GA	466.15 (324.4-659.1), 20	489.95 (357.6-699.6), 66	0.543
	AA	481.9 (382.4-688), 13	404.8 (359.8-633.5), 18	0.650
	$P_{Kruskal-Wallis}$	0.861	0.570	
Homocysteine µmol/L	GG	14.1 (12-17.5), 11	13.0 (11.2-14.8), 57	0.424
	GA	16.6 (11.4-18.1), 30	14.3 (11.3-18.3), 95	0.642
	AA	16.25 (14.08-19.8), 18	13.0 (11.3-16.5), 31	0.081
	$P_{Kruskal-Wallis}$	0.681	0.411	
	Males			
Serum folates nmol/L	GG	18.0 (12.45-23.25), 24	15.2 (12.7-22.4), 57	0.706
	GA	17.5 (11.8-22.2), 37	17.35 (12.7-22.3), 96	0.984
	AA	21.95 (16.3-25.6), 10	17.5 (13.4-22), 59	0.143
	$P_{Kruskal-Wallis}$	0.373	0.708	
RBC folates nmol/L	GG	578.2 (517.3-723.65), 20	494.9 (383.5-781.7), 41	0.348
	GA	549.85 (430.1-681.45), 24	507.5 (392.3-642.2), 74	0.490
	AA	596.4 (362.5-785.3), 8	483.3 (349.5-621.6), 41	0.386
	$P_{Kruskal-Wallis}$	0.777	0.680	
RBC folates corrected nmol/L	GG	545.6 (487.1-702.75), 20	459.75 (361.85-723.85), 40	0.315
	GA	528.5 (416.95-646.8), 24	488.4 (367.7-614.7), 74	0.490
	AA	563.95 (330.7-755.8), 8	457.7 (338.1-602), 41	0.464
	$P_{Kruskal-Wallis}$	0.813	0.622	
Homocysteine µmol/L	GG	15.6 (11.1-19.2), 23	17.1 (13.2-20), 57	0.473
	GA	16.1 (11.2-19.4), 37	15.0 (12.35-18.6), 96	0.675
	AA	12.9 (11.7-15.8), 10	16.3 (12.7-21.2), 59	0.049
	$P_{Kruskal-Wallis}$	0.650	0.334	
